# The Impact of Iron Chelators on the Biology of Cancer Stem Cells

**DOI:** 10.3390/ijms23010089

**Published:** 2021-12-22

**Authors:** Julia Szymonik, Kamila Wala, Tomasz Górnicki, Jolanta Saczko, Bartosz Pencakowski, Julita Kulbacka

**Affiliations:** 1 Faculty of Medicine, Wroclaw Medical University, 50-367 Wroclaw, Poland; julia.szymonik@student.umw.edu.pl (J.S.); kamila.wala.01@gmail.com (K.W.); tomasz.gornicki@student.umw.edu.pl (T.G.); 2 Department of Molecular and Cellular Biology, Faculty of Pharmacy, Wroclaw Medical University, 50-556 Wroclaw, Poland; jolanta.saczko@umw.edu.pl; 3 Department of Pharmaceutical Biology and Botany, Faculty of Pharmacy, Wroclaw Medical University, 50-367 Wroclaw, Poland; bartosz.pencakowski@umw.edu.pl

**Keywords:** cancer stem cells, stemness markers, iron chelators, deferoxamine, deferasirox

## Abstract

Neoplastic diseases are still a major medical challenge, requiring a constant search for new therapeutic options. A serious problem of many cancers is resistance to anticancer drugs and disease progression in metastases or local recurrence. These characteristics of cancer cells may be related to the specific properties of cancer stem cells (CSC). CSCs are involved in inhibiting cells’ maturation, which is essential for maintaining their self-renewal capacity and pluripotency. They show increased expression of transcription factor proteins, which were defined as stemness-related markers. This group of proteins includes OCT4, SOX2, KLF4, Nanog, and SALL4. It has been noticed that the metabolism of cancer cells is changed, and the demand for iron is significantly increased. Iron chelators have been proven to have antitumor activity and influence the expression of stemness-related markers, thus reducing chemoresistance and the risk of tumor cell progression. This prompts further investigation of these agents as promising anticancer novel drugs. The article presents the characteristics of stemness markers and their influence on the development and course of neoplastic disease. Available iron chelators were also described, and their effects on cancer cells and expression of stemness-related markers were analyzed.

## 1. Introduction

Despite tremendous advances in medicine over the years, effective anticancer treatment is still a therapeutic challenge. A growing problem in the successful neoplasm treatment is the chemoresistance of cancer cells [1]. Multi-drug resistance requires a search for new clinical solutions due to the inability to continuously increase the use of chemotherapeutic agents. Resistance to anticancer drugs may be related to the specific properties of cancer stem cells (CSCs) [2]. Expression of the transcription factor proteins, defined as stemness-related markers, was demonstrated in CSCs [3]. These cells are involved in inhibiting the maturation of CSCs, which is essential for maintaining their self-renewal capacity and pluripotency [4]. Successful inhibition of the expression of the stemness-related markers was confirmed to be associated with a reduction in resistance to chemotherapeutic agents and decreased tumor growth and CSCs migration. It has been shown that the metabolism of cancer cells changes, and the demand for iron significantly increases. The proper expression of genes, including genes encoding stemness-related markers, requires constant access to an adequate iron level [5]. Therefore, a thesis has been made that reducing the availability of this element by chelating substances will decrease the expression of stemness markers, thus causing CSCs to lose the ability of self-renew and a decrease resistance to chemotherapy.

The article presents the stemness markers along with their characteristics and influence on the development and course of neoplastic disease. Available iron chelators were also described, and their effects on cancer cells and expression of stemness-related markers were analyzed.

## 2. Impact of Iron Chelators on the Cancer Cells

Iron metabolism in neoplastic cells is modulated in several ways to cover the increased iron requirements. Cancer cells enhance iron uptake by the overexpression of transferrin receptors 1 and 2 (TfR1, TfR2), which are responsible for the internalization of iron complexed with transferrin (Tf) [6]. Increased expression of c-Myc was found to be correlated with decreased expression of ferritin affecting the labile iron pool and increased level of iron regulatory protein 2 (IRP2) that further upregulates TfR1 [7]. Iron chelators, with their ability to sequester this pivotal metal, can influence the cancer cell metabolism in multiple ways, mostly concentrated around inducing apoptosis and cell cycle arrest. Iron depletion causes cell cycle inhibition by the downregulation of cell cycle-related proteins such as cyclin D1, A, B, and cyclin-dependent kinase 2 (cdk2). On the other hand, expression of protein p53 and n-Myc downstream-regulated gene-1 (Ndrg1) involved in pathways related to apoptosis increases in the absence of iron. Proteins belonging to c-Jun amino-terminal kinase (JNK) and p38 signal transduction pathways taking part in the cell cycle arrest are also upregulated [6]. Other important points of cancer cell biology attacked by low iron level are the modulation of histone methylation, decreased level of ribonucleotide reductase (RR), and remodeling of the cancer microenvironment [8]. Some studies also imply that chelators have the ability to increase the production of ROS in cancer cells, increasing oxidative stress [9].

## 3. Stemness-Related Markers

CSCs are a crucial subpopulation of tumor cells due to their unique properties of self-renewal ability and multilineage differentiation potential [5]. CSCs are essential in the process of tumor initiation and play a significant role in the formation of metastases, relapse, and chemoresistance [10]. CSCs are found to express markers that are manifested in embryonic or adult stem cells, while these markers are not present in normal somatic cells. As a consequence, cells gain a ‘stemness’ phenotype. Nowadays, nearly 25 transcriptional factors (TFs) have been discovered to be expressed in stem cells. Among them, a group creates a core regulatory network for embryonic stem cells (ESCs) maintenance and self-renewal. This group contains OCT4, SOX2, KLF4, Nanog, and SALL4, highly expressed in ESCs and suppressed in normal somatic cells. Numerous evidence revealed that embryonic specific TFs are abnormally expressed in human tumor samples, suggesting the stemness features. Currently, the presence of CSCs in cancer or cell culture could be confirmed by detecting three or more markers, at least one of which is specific for the given type of cancer [11]. Table 1 shows the stemness-related markers and their primary function in stem cells and the types of neoplasms in which these markers are present and negatively affect the prognosis.

### 3.1. SOX2

SRY-Box Transcription Factor 2 (SOX2) is a stemness-related transcriptional factor in charge of stem cell self-renew and pluripotency maintenance [5]. In the human body, the SOX2 gene is located on chromosome 3 at position q26.3–27. This gene encodes a protein of 317 amino acids, which could be modified through phosphorylation, acetylation, methylation, etc. As a result of these modifications, SOX2 displays different activities [29]. The beginning of expression SOX2 occurs very early in embryonic development and plays a crucial role in aggregating the pluripotent inner cell mass-the lack of SOX2 leads to embryonic lethality [30]. In pluripotent stem cells, the retention of stemness is reached by the so-called core transcriptional network consisting of SOX2 and the transcription factors: OCT4 and Nanog. These three factors cooperate functionally to promote the expression of pluripotency-associated genes (Nanog, OCT4, SOX2, among others) and suppress the expression of genes associated with differentiation [31]. The expression of SOX2 can be detected in embryonic neural progenitor cells (NPCs) and persists in adult neural stem cells and NPCs in several neurogenic regions. In addition, mesenchymal stem cells (MSCs) have been found to rely on SOX2 expression to maintain stemness, proliferation, and proper differentiation. Deregulation of SOX2 expression is an important agent promoting the pathogenesis of cancer. SOX2 is responsible for some characteristics of cancer cells such as proliferation, epithelial–mesenchymal transition (EMT), migration, invasion, metastasis, spherical and colony formation, tumor initiation, cancer stem cell formation, and resistance to apoptosis and therapy [32]. SOX2 is found to be expressed in tumors of the brain, breast, lung, liver, prostate, testis, and seminoma [33,34]. The expression of SOX2 correlates with poor prognosis for stage I lung adenocarcinoma, squamous cell carcinoma, gastric carcinoma, small cell lung cancer, and ovarian carcinoma [35,36]. In the primary tissue of head and neck squamous cell carcinoma (HNSCC) patients, expression of SOX2 is abnormal but stays standard in healthy tissue [37]. Investigation of HSNCC-represented by line SNU1041-revealed that overexpression of SOX2 hastens the formation of sphere cells, which are the hallmark of self-renewal properties [38].

In addition to that, knocking down SOX2 in HNSCC CSCs deprives them of their self-renewal ability, chemoresistance (resulting from ABCG2 suppression), invasion capacity, and tumorigenicity in vivo [38]. In addition, SOX2 and OCT4 expression is positively related to tumor aggressiveness in breast cancer [39].

### 3.2. OCT4

Octamer-binding transcription factor 4 (OCT4) is a homeodomain transcription factor of the POU family and plays an essential role in regulating pluripotency during embryogenesis and tumorigenesis by managing pluripotency self-renewal abilities. In normal mature organism cells, the OCT4 is undetectable, but its re-expression is highly connected with tumor growth and progression of cancer [40]. The correlation between OCT4 and the occurrence of CSCs was confirmed in various types of cancers, including melanoma, prostate cancer, and hepatoma [41]. OCT4 expression increases as the degree of cell differentiation decrease and remains highest for undifferentiated cells. It has been revealed that cells that are chemotherapy-resistant present higher levels of OCT4 than chemotherapy-sensitive ones [11]. The research results revealed that OCT4 expression is correlated with poor clinical prognosis in hormone receptor-positive breast cancer, bladder cancer, and adenocarcinoma of the lung [39]. Overexpression of OCT4 induces the activation of TCL1, AKT, and ABCG2, mediating the process of chemoresistance [42]. According to these facts, we might suspect that resistance to chemotherapy is connected with OCT4 expression level. OCT4 expression is responsible for maintaining the properties of stem cells in lung cancer cells. It is suggested that TNF-α, IL-1β, or IL-6 promote the expression of the OCT4 marker. Lung cancer cells that overexpress OCT-4 secrete a macrophage colony-stimulating factor (M-CSF), which stimulates tumor cells’ macrophage-mediated migration through blood vessels. This combination of factors positively corresponds to the cancer relapse [41].

Moreover, lung cancer cells that overexpress OCT4 remain resistant to chemotherapeutic agents used in conventional therapy, such as cisplatin, doxorubicin, and etoposide [43]. These cells are also not sensitive to targeted therapy with gefitinib [44]. The correlation between increased OCT4 and SOX2 expression and cisplatin resistance also occurs in mesothelioma cells [16]. CSCs are considered to be a cause of resistance to the standard chemotherapy used in pancreatic cancer. In these cells, CD44 is overexpressed. This state of affairs results in SOX2, OCT4, and Nanog upregulation, which leads to intensified clone formation and cell growth and self-renewal [45].

Human hepatocellular carcinoma (hHCC) is highly aggressive, resulting from acquiring the features of stem cells [46]. OCT4 expression promotes hHCC progression and plays a crucial role in this process [47]. In addition, in the case of hHCC and head and neck cancer, the upregulation of OCT4 is responsible for resistance to chemotherapy [42]. Compared to control cells, a higher proliferation rate and higher resistance to hypoxia and cisplatin are found in melanoma cells with exogenous OCT4 expression [48].

The expression level of OCT4, SOX2, and Nanog remains high in glioblastoma-derived circulating tumor cells, making them resistant to temozolomide and gamma radiation therapy [49]. The OCT4 knockdown reduces the stemness properties of germ cell tumors and increases the sensitivity to cisplatin and irradiation used to treat lung and ovarian cancer [50]. Furthermore, if OCT4 is knocked out, the proliferation rate of HCC decreases and the EMT process becomes inverted.

### 3.3. Nanog

Homeobox protein Nanog is a stem cell transcriptional factor initially described in May 2003 [51]. Nanog plays a focal role in embryonic development, maintaining pluripotency and being the downstream target of other pluripotency-related genes like SOX2 or OCT4. The role of Nanog in embryonic development was found to be limited not only to the early stages of development. Studies indicate the role of this transcription factor in the development of ovary, testes, and tooth germ cells [52]. Expression of Nanog has also been proven in cardiac mesenchymal stem cells [53]. The importance of Nanog in the postnatal stage of a healthy human organism decreases significantly with an expression on mRNA or protein level found in only few organs, for example, the thyroid gland, the small intestine, and the glandular cells of the uterine cervix. Considering the role of Nanog in the regulation of stem cells differentiation, the role of this transcription factor in the process of carcinogenesis seems obvious. Numerous studies proved this statement to be correct. Nanog expression was very important in many different types of cancer, such as gastric, rectal, breast, and pancreatic cancer. It can act as both a predictive or prognostic marker, but there is evidence indicating that inhibition of Nanog inhibits stem-like properties of cancer cells [54]. There is strong evidence that Nanog plays a role in maintaining CSC phenotype. Increased Nanog level caused by hypoxia stimulated hypoxia-inducible factor (HIF)-1α and HIF-2α increases the percentage of breast cancer stem cells [55]. ERK1/2-Nanog signaling pathway enhances the stem-like phenotype of cells and epithelial to mesenchymal transition in HNSCC [56]. An available study also showed that Nanog overexpression mediates tobacco smoke-induced stem-like phenotype in renal cancer [20].

### 3.4. c-Myc

c-Myc, n-Myc, and l-Myc are fundamental transcription factors encoded by the proto-oncogene family. During the normal process of embryogenesis, c-Myc and n-Myc factors play a significant role as a regulator because the Myc family is essential for acquiring and maintaining the stem-cell qualities. These properties give the cell the ability to self-renewal and differentiation into multiple lines [57]. A wide range of services provided by c-Myc includes its role in cell differentiation, growth, and division. This TF is also involved in genome stability and angiogenesis [58]. It is also worth mentioning that c-Myc is involved in the regulation of carcinogenesis and progression in various types of cancer [59]. Furthermore, c-Myc stimulates malignant transformation and has been found to be essential for cancer stem cell properties [57,60]. In general, c-Myc is expressed in multiple adult tissues, while its constitutive upregulation has been noticed in carcinoma of the colon, cervix, lung, stomach, and breast [60]. Amplification and increased c-Myc oncogene expression are strongly correlated with resistance to drugs used in cancer therapy [61]. c-Myc’s likely mechanism of action contributing to drug resistance includes increased expression of genes that promote cell survival, inhibit apoptosis, and destabilize the genome [58].

Cancer cells may also acquire c-Myc-mediated resistance to chemotherapy if they previously received cisplatin [62]. In HNSCC, prior treatment with cisplatin may lead to increased resistance to Palbociclib through a mechanism associated with increased c-Myc expression. As proof of the c-Myc up-regulation in the acquisition of cancer cells resistance to Palbociclib, it is reported that the simultaneous treatment with the c-Myc inhibitor JQ1 and Palbociclib resulted in a synergistic anti-tumor growth effect [63]. Overexpression of c-Myc in human breast cancer cells could induce EMT [64].

The EMT is a critical process in which cells change their phenotypes from epithelial toward more motile phenotypes with a higher ability to invade the surrounding tissue and migrate. Consequently, EMT is highly connected with chemoresistance and the occurrence of metastasis [65]. In urothelial carcinoma cell lines, the expression of c-Myc is correlated with the expression of CD44, an integral cell membrane glycoprotein involved in cell migration and tumor cell invasion and metastasis. Furthermore, in these cell lines, Kaplan–Meier survival analysis showed a strong correlation between overexpression of c-Myc and poor recurrence-free survival [23]. n-Myc and c-Myc are strongly interrelated in the regulation of the neuroblastoma cancer stem cell phenotype and radio-resistance upon glutamine deprivation. Glutamine deprivation with radiotherapy in n-Myc-amplified neuroblastoma brings on the increased number of radioresistant cells that is associated with up-regulation of c-Myc in these cells [22]. To sum up, as c-Myc is an essential regulator of survival and growth of cancer, it is suggested that the c-Myc protein may be a potential molecular target for cancer therapy.

### 3.5. KLF4

Krüppel-like factor 4 (KLF4) is an evolutionary conservative transcription factor with zinc finger motif [66]. The corresponding gene is located on chromosome 9. KLF4 belongs to SP/KLF family and contains 483 amino acids [67]. This factor has a major role in the evolution and functioning of many different tissues and organs. It maintains homeostasis of intestinal epithelium by being a focal point of regulation in the development of goblet cells and enterocytes [68]. KLF4 is responsible for regulating the position of Paneth cells and is a critical network regulator regulating corneal homeostasis [69]. In skin, KLF4 was found to be responsible for epidermal differentiation, the creation of epidermal barrier, and promoting wound healing [70]. Other physiological functions of KLF4 include regulating spermatogenesis, maintaining the integrity of the endothelial barrier, and reducing the inflammatory response in kidneys [71].

KLF4 plays an important role in physiology and several pathological processes like inflammation and radiation injury that outreach the subject of this review, but most notably in the development and progression of cancer. KLF4 is a transcriptional factor whose role in carcinogenesis is context-dependent, being upregulated or downregulated in different types of cancer. Loss of KLF4 expression is frequent in gastric cancer, colorectal cancer, intestinal cancer, bladder cancer, and lung cancer, acting as a tumor suppressor gene [72]. On the other hand, high expression of KLF4 is crucial for maintaining breast cancer stem cells [73]. Increased expression was also found in HNSCC [74]. In the skin, overexpression of KLF4 leads to dysplasia and eventually squamous cell carcinoma [75]. In 2006, KLF4 was described as a stemness factor for the first time. There was proven that KLF4 overexpression induced the dedifferentiation of adult mouse fibroblasts into pluripotent stem cells [76]. The fact that KLF4 is stemness factor suggests the association of KLF4 with cancer stem cells. That correlation turned out to be correct and supported by studies showing the role of KLF4 in promoting and maintaining cancer stem cells. Osteosarcoma cells that overexpressed KLF4 displayed characteristics of cancer stem cells [24]. KLF4-DYRK2 pathway regulates the self-renewal of stem cells in chronic myeloid leukemia [25]. Another study on hepatocellular carcinoma revealed the ability of KLF4 to turn noncancer stem cells into cancer stem cells [26].

### 3.6. SALL4

In humans, the Sal-like protein 4 (SALL4) gene is located in 20q13.2 and belongs to the Spat-Like gene family [77]. SALL4 is transcription factor with multiple Cys(2)his(2) (C2H2)-type zinc finger domains in its structure [78]. SALL4 occurs as an essential part of inner cell mass proper development during mice development [79]. Researchers also provide evidence of SALL4 expression in the midbrain, left ventricular myocardium, etc., [80]. Expression of SALL4 in ovary and testis in adult human tissue is almost identical to this one found in mice, with one exception being human CD34+ hematopoietic stem/progenitor cells. What is more, SALL4 turned out to be also essential for pluripotency of ESCs [80]. There is also evidence of SALL4 in the generation of iPS cells from fibroblasts [81]. Defects of SALL4 are connected to multiple autosomal dominant diseases such as Okihiro/Duane-radial ray syndrome, acro-renal-ocular syndrome, and Instituto Venezolano de Investigaciones Cientificas syndrome [82]. Deregulation of SALL4 was found in many different types of cancer such as leukemia, glioma, breast cancer, germ cells tumor, and colorectal cancer. SALL4 is responsible for the transformation, survival, metastasis, and drug resistance of cancer cells. SALL4 turned out also to be essential for CSCs self-renewal [83]. Likewise, overexpression of SALL4 allows gastric cancer cells to acquire stem cell properties [68].

## 4. Iron Chelators

Iron metabolism in tumor cells is altered. Cancer cells have a huge need for high intracellular iron concentrations due to their rapid self-metabolism and proliferation. This requirement is manifested by high ferritin levels (light and heavy chain), the protein responsible for iron storage [84]. Iron chelators are small natural–derived from microorganisms (siderophores)-or synthetic particles that can decrease the intracellular level of iron. The group of iron chelators includes compounds such as 3-AP, SIHA, DFO, DFX, Dp44mT, EP, CPX, which show the ability to sequester metals necessary for tumor growth, and due to that fact, there is increasing interest in exploring their potential anticancer properties [85]. Among them, deferoxamine (DFO) and deferasirox (DFX) are approved by FDA in the treatment of iron overload, which is secondary to repeated blood transfusions in patients with leukemia [3]. The mode of action of chelators is based on proliferation inhibition, apoptosis and differentiation induction, and inhibition of ribonucleotide reductase. Iron chelators also induce cell cycle arrest and ROS generation [86].

### 4.1. Siderophores

Siderophores are low-molecular-mass Fe^3+^-specific chelators responsible for iron uptake and storage. It has been noted that iron-dependent microorganisms synthesize siderophores under iron stressed conditions to reduce iron toxicity [87]. There are a few types of siderophores, which differ in chemical structure and properties [88]. Some of them are presented and discussed below.

#### Deferoxamine (DFO)

Deferoxamine is one of the most widely used iron chelators. It is highly effective in removing iron and has low toxicity. Clinically, it is used to treat chronic iron overload in the body, e.g., in post-transfusion hemosiderosis. In addition to iron, DFO can chelate other molecules such as aluminum. It is administered by subcutaneous infusions at a dose of 40–60 mg/kg for 8–12 h, 3–5 times a week. Intravenous or, rarely, intramuscular injections are also possible [89]. The antioxidant potential of DFO was also noted, which appears to be independent of the chelating capacity of DFO and can be used in the treatment of other pathologies. Iron chelators have a beneficial effect on tissue regeneration due to their angiogenic effect and reduced damage caused by reactive oxygen species (ROS) [90]. In an in vivo study on a hypoxic-damaged neonatal mouse brain model, a neuroprotective effect of DFO was demonstrated. The exact mechanism of action is not fully understood. However, DFO can increase hypoxia-inducible factor-1 alpha (HIF-1a), which in turn increases VEGF levels and promotes angiogenesis [91]. It has also been proven that DFO protects bone marrow-derived mesenchymal stem cells from apoptosis by causing iron deficiency and reducing oxidative stress [92]. Numerous studies have focused on identifying the potential benefits of iron chelators in oncology. DFO reduces the chemoresistance of cancer cells, thereby increasing the anticancer effect of drugs, such as cisplatin, in the treatment of ovarian cancer [84]. The reduced iron concentration by iron chelators inhibits the proliferation of human lymphocytes B and T. The mechanism of action is to reduce ribonucleotide reductase activity, thus inhibiting DNA synthesis and cell proliferation. This process is reversible, the supply of Fe^3+^ restores the normal cell cycle, which confirms the important role of this microelement in the described mechanism of lymphocyte proliferation inhibition [93]. Furthermore, in clinical trials, the administration of DFO (5-day course at 150 mg/kg/day) to patients with neuroblastoma reduced bone marrow infiltration in seven out of nine patients without causing toxic effects [94]. Other clinical trials (NCT03652467) are currently underway to investigate the safety and efficacy of DFO in patients with unresectable hepatocellular carcinoma.

### 4.2. Synthetic Chelators

#### 4.2.1. DFX

Deferasirox (DFX) is an orally administered drug to treat iron overload, most often as a second-line drug in the case of DFO intolerance. Oral administration of DFX once a day at a dose of 30–40 mg/kg/day is more convenient to use. However, this novel agent’s long-term effectiveness and safety information are more limited than when compared to DFO [95]. This chelator effectively reduces iron levels in patients with β-thalassemia major or post-transfusion secondary iron overload in both adult and pediatric patients [96]. However, it is worth mentioning that cases of severe toxicity following the administration of DFO have been reported. The primarily renal or hepatic failure occurred in patients treated with DFX [97]. Other side effects such as Fanconi syndrome and bone marrow damage were also seen [98]. This damage mechanism is not fully understood, but the most probable seems to be the toxic effect of DFX on mitochondria, the formation of ATP, and thus the disturbance of the oxygen metabolism of cells [99].

Similar to DFO, also in the case of DFX, its potential anticancer effect was investigated. Iron chelator DFX has been shown to potentiate the effects of standard chemotherapeutic agents such as carboplatin, doxorubicin, or cisplatin in triple-negative breast cancers [100]. The above-mentioned anti-tumor action is based on inhibiting the proliferation of tumor cells and the induction of apoptosis. Both DFO and DFX suppress the growth of esophageal cancers in two esophageal adenocarcinoma cell lines, the squamous esophageal cell line and in vivo studies with murine xenograft model. Chelating agents decreased intracellular iron levels and thus reduced cell viability and proliferation of cancer cells. Furthermore, iron chelators showed the ability to overcome chemoresistance, e.g., to cisplatin [101]. Oral iron chelator also inhibits pancreatic cancer proliferation in a dose-dependent manner. It has been shown that 10 µM DFX causes cell cycle arrest in the S phase, while 50 and 100 µM DFX leads to apoptosis. The suppression of neoplastic cells resulted from reduced iron availability and was associated with the downregulation of transforming growth factor-ß1 [102]. Recently, research has revealed that DFX can also inhibit pancreatic cancer cells’ invasion by reducing Cdc42 and Rac1 activation [103].

#### 4.2.2. Dp44mT

The iron chelator Dp44mT is an anticancer agent which increases the p-AMPK/AMPK ratio in multiple tumor cell types [104]. The enzyme 5′-adenosine monophosphate-activated protein kinase (AMPK) plays a role in pathways involving apoptosis. Dp44mT activates the AMPK-dependent stress pathway in a variety of tumor cell types. This up-regulation of the AMPK pathway mediated by Dp44mT was also revealed to inhibit fatty acid and protein biosynthesis. All the above leads to the increased activity of regulators and downstream catabolic pathways, such as autophagy in cancer cells [105]. In medulloblastoma cell lines cultured in vitro with Dp44mT the sphere size and its stemness ability were significantly decreased [106]. In vivo Dp44mT inhibits metastasis of HCC by suppressing EMT via n-Myc downstream-regulated gene 2 [107]. In acute lymphoblastic leukemia, Dp44mT induces apoptosis and cell cycle arrest at the G1/S stage [3]. Cell cycle arrest induced by Dp44mT is a mechanism that leads to tumor growth arrest. In breast cancer cells, Dp44mT causes DNA damage and selective inhibition of top2α activity. Dp44mT could act as a substance with a synergistic effect to doxorubicin at low concentrations by enhancing its cytotoxic effect on breast cancer cells [108].

#### 4.2.3. Other Iron Chelators

Other chelators such as 3-AP, CPX, EP, SIHA show their unique mode of action in leukemia treatment. For example, SIHA displayed the ability to induce apoptosis, cell cycle arrest, and dissipation of the mitochondrial membrane, while EP-the thrombopoietin receptor agonist-induces differentiation and G1 cell cycle arrest [3]. These chelators expressed their anticancer effects in acute myeloid leukemia (AML). Notwithstanding their proven mode of action, these chelators also require further investigation about their effect on the expression of stemness-related markers in CSC.

## 5. Impact of Iron Chelators on CSC

**Figure 1:**
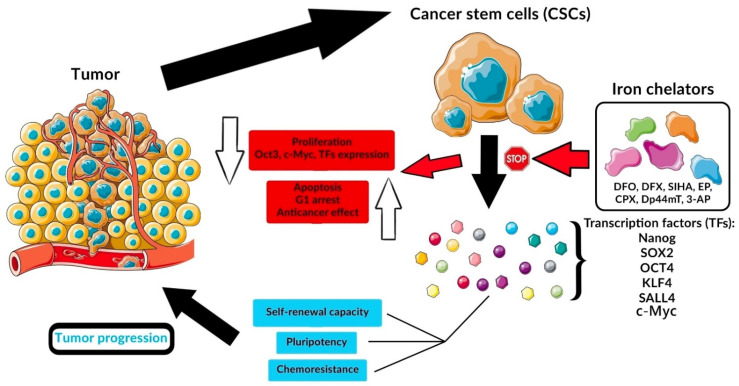
Effect of various iron chelators on CSC and tumor progression.

The proven antitumor activity of chelating agents prompted researchers to further investigations to elucidate their exact mechanism of action. A new direction of research is studying the effect of iron chelators on markers of CSCs. Figure 1 shows the effect of iron chelators on tumor stem markers and thus tumor progression.

Since DFO and DFX are the most commonly used chelators, the effect on CSC was studied mainly for these substances. Dp44mT and other chelators require more detailed research on their effect on expressing stemness-related markers in CSC. Both DFO and DFX inhibited the proliferation of miPS-LLccm cells, used as a CSC model. Additionally, the expression of stemness markers including Nanog, SOX2, c-Myc, OCT3/4, and KLF4 also decreased. Interestingly, after using traditional anticancer drugs, such as cisplatin, suppression of the tumor was noticed. However, there were no effects on stemness-related markers [109]. It has been shown that DFO downregulates the expression of cancer stem cell markers, including SOX2, Nanog, and c-Myc in two ovarian cancer cell lines (SKOV-3 and OVCAR-3) [84]. DFX has also been proved to be effective in altering the expression of stemness markers. This iron chelator inhibited the expression of Nanog, c-Myc, SOX2, OCT3/4, and KLF-4 in four cancer cell lines (HSC-2: Oral squamous cancer, TE-4, and OE33: Esophageal cancer, and NT-2: Embryonal cell carcinoma) [110,111]. At the same time, a decreased sphere formation ability of cancer cells was noticed [111].

Katsura et al. investigating the exact mechanism of action of iron chelators found that DFX suppresses the Stat3 signaling pathway, which is related to the expression of Nanog [112]. In turn, Narusaka et al. suspect that the effect of iron chelators on the stemness of cancer cells is due to the induction of reactive oxygen species generation by DFO and DFX [113]. Importantly, DFX exhibits a specific effect on tumor cells with high expression of stem markers. Furthermore, studies on human fibroblast (non-cancerous) cells (WI38, FEF3) showed only minimal toxicity of DFX, which indicates the selectivity of this substance towards cancer cells [112].

In esophageal cancer, high Nanog expression correlates with chemoresistance to conventional, neoadjuvant therapy and leads to poor overall survival. The studies of Narusaka et al. showed a high expression level of stemness markers such as Nanog, c-Myc, or KLF4 in esophageal squamous cancer cell lines (TE8) and adenocarcinoma cell lines (OE33). Standard chemotherapy not only maintains the current expression level of the stemness markers but also may increase it slightly; in comparison, iron chelators can lower it. After culturing TE8 and OE33 cells with different concentrations of DFX for 48 h, most stemness markers were suppressed. Furthermore, iron chelators also suppress cancer cell proliferation. Moreover, in contrast to CDDP (cisplatin) causing the compensatory release of the inflammatory cytokine Il-6, which worsens the prognosis, the use of iron chelators in therapy does not induce interleukin secretion. Overall, inhibition of the compensatory IL-6 secretion and decreases of stemness caused by iron chelators may become a novel therapeutic strategy for esophageal cancer [110].

A recent study conducted by Fiorillo et al. revealed that Deferiprone (DFP) inhibits propagation of breast cancer CSC by targeting mitochondrial metabolism, glycolysis, and increasing production of ROS. The authors showed a significant reduction in crucial cell parameters like mitochondrial oxygen consumption (OCR) and extracellular acidification rates (ECAR), providing data to explore another field of chelators’ impact on CSC biology. It is very important to notice that tested concentrations of DFP work in a dose-dependent manner and haven’t affected normal cell lines. Table 2 summarizes information on iron chelators, including their effects on cancer cells and CSC phenotype.

## 6. Summary

Iron metabolism is crucial for cancer cells. Iron chelators show anticancer activity and can be a potential novel agent in oncological treatment. A promising therapeutic target appears to be CSCs and their stemness-related markers. Thus far, the effectiveness of two FDA-approved chelators, DFO and DFX, in suppressing the stemness of cancer cells has been confirmed. Therefore, iron chelators could be used as an additional therapeutic option, especially in cases of high expression of stemness-related markers in high-grade, recurrent, and rapidly metastatic cancers. The widespread use of these chelating agents and numerous studies on the safety of their use provide a real possibility of their clinical use in cancer patients as a standard treatment. However, further research is needed to determine the effect of other chelators on tumor cell stem markers and their potential utility in cancer treatment.

## Figures and Tables

**Table 1:** Characteristics of stemness-related markers, including their function, localization of expression, and types of neoplasms in which markers deteriorate prognosis.

Marker	Function in Stem Cell	Expressed in Tumor Types	Poor Prognosis for Tumor Types	References
SOX2	Stem cell self-renew and pluripotency maintenance	Breast, colorectal, oral SCC, glioblastoma, esophageal, nasopharyngeal, prostate, ovarian, non-small cell lung, head, and neck SCC, liver, testis.	Stage I lung adenocarcinoma Esophageal squamous cell carcinoma Breast cancer Squamous cell carcinoma Gastric carcinoma Prostate cancer Small cell lung cancer Ovarian carcinoma	[12,13,14]
OCT4	Stem cell self-renew and pluripotency maintenance	Bladder, brain, breast, cervical cancer, oral squamous cell carcinoma, hepatocellular carcinoma, lung cancer, leukemia, ovarian, mesothelioma, pancreas, prostate, renal, seminoma, testis.	Bladder cancer Esophageal squamous cell carcinoma Medulloblastoma Prostate cancer	[15,16,17]
Nanog	Stem cell self-renew and pluripotency maintenance	Breast, Gastric, Brain, Pancreatic, Prostate, colon, renal, liver, Ovarian, germ cell tumors	Breast cancer Colorectal cancer Gastric adenocarcinoma Liver cancer Non-small cell lung cancer Ovarian Serous Carcinoma	[5,18,19,20]
c-Myc	Stem cell self-renewal	Cervix, Testis, Lymphoma, Leukemia, Stomach, Breast, Colon, Myeloma, Lung, Brain, Head and Neck, Pancreas, Prostate, Renal, Salivary-gland, Urothelial carcinoma, Neuroblastoma	Early carcinoma of the uterine cervix Hepatocellular carcinoma	[21,22,23]
KLF4	Stem cell self-renew and pluripotency maintenance	Osteosarcoma, Leukemia, Myeloma, Colon, Hepatocellular carcinoma, Brain, Breast, Head and Neck, Oral, Prostate, Testis	Breast cancer Nasopharyngeal carcinoma Colon cancer Head and neck squamous cell carcinoma Oral cancer	[24,25,26]
SALL4	Stem cell self-renew and pluripotency maintenance. Differentiation regulation	Leukemia, Glioma, Breast, Liver, Colorectal cancer, Ovarian, Testis, Hepatocellular carcinoma	Hepatocellular carcinoma Gliomas Myelodysplastic syndromes	[27,28]

**Table 2:** Summary of iron chelators and their effect on cancer cells and stemness-related markers.

Iron Chelator	Substance	Effectiveness in Oncology	Impact on CSC Phenotype	References
Siderophores	Deferoxamine (DFO)	DFO meaningly inhibits the proliferation of leukemia cells. Deferoxamine induces apoptosis in ovarian cancer, and its cytotoxicity is dose- and time-dependent. Desferrioxamine and cisplatin may act as a new effective combined therapy in ovarian cancer.	In ovarian cancer stem cells, the expression of cancer stem cell markers such as SOX2, Nanog, and c-Myc is decreased after treatment with DFO. DFO significantly inhibits the expression of stemness-related markers such as Nanog and OCT3/4 in miPS-LLCcm cells.	[84,109]
Synthetic chelators	Deferasirox (DFX)	In AML and ALL DFX exerts antileukemia activity by inhibiting extracellular signal-regulated kinase (ERK) phosphorylation, repressing the mammalian target of rapamycin (mTOR) and NF-κB signaling pathway. In vitro administration of DFX inhibits the proliferation of the hepatoma cell line and induces caspase-3 activation in a dose-dependent manner. DFX inhibits the proliferation of pancreatic cancer cells in a dose-dependent manner. Also, in pancreatic cancer, the cells treated by DFX had significantly reduced invasive ability compared with control cells. Deferasirox effectively depletes iron from esophageal tumor cells, which results in the suppression of cancer growth in vitro and in vivo.	DFX inhibits the proliferation and expression of stemness markers such a Nanog, SOX2, OCT3/4, KLF4, c-Myc in the human cancer cell lines in a dose-dependent manner. Western blot analysis of miPS-LLCcm cells showed that deferasirox significantly suppresses expression of the stemness markers Nanog, OCT3/4, SOX2, c-Myc and KLF4 in a dose-dependent manner.	[3,103,112]
	Dp44mt	In a human colon cancer cell line HT-29 and human prostate cancer cell line DU145 the Dp44mT inhibits TGF-β-induced Epithelial-Mesenchymal Transition via Up-Regulation of NDRG1. In glioma cells, Dp44mT induces apoptosis via RORA-mediated NDRG2-IL6/JAK2/STAT3 signaling, leading to tumor growth suppression. Both in vitro and in vivo Dp44mT suppresses osteosarcoma proliferation migration and invasion.	not yet established	[114]
	Other chelators	EP induces differentiation and cell cycle arrest at the G1 stage. SIHA induces apoptosis, cell cycle arrest and dissipation of the mitochondrial membrane potential 3-AP, CPX act as ribonucleotide reductase inhibitors	not yet established	[3]
